# Primary CD5^+^ nongerminal center B-cell diffuse large B-cell lymphoma of the breast: A case report

**DOI:** 10.1016/j.jdcr.2026.08.003

**Published:** 2026-08-17

**Authors:** Peyton Grant, Allison Goldenstein, David Cleaver, Jonathan Cleaver

**Affiliations:** a Kirksville College of Osteopathic Medicine, A.T. Still University, Kirksville, Missouri; b Northeast Regional Medical Center, A.T. Still University, Kirksville, Missouri; c Cleaver Dermatology, Kirksville, Missouri

**Keywords:** CD5^+^, diffuse large B-cell lymphoma (DLBCL), nongerminal center B-cell (non-GCB), primary breast lymphoma

## Introduction

Primary breast lymphoma (PBL) is an extranodal non-Hodgkin lymphoma that originates within breast tissue in patients without a previous history of systemic lymphoma. PBL is rare, accounting for ∼0.04% to 1% of all malignant breast neoplasms and <1% of all non-Hodgkin lymphomas.^1^, ^2^, ^3^ Diffuse large B-cell lymphoma (DLBCL) is one of the most common and histologically aggressive subtypes of PBL.^1^, ^2^, ^3^ The rarity of PBL and its clinical similarity to other breast tumors continue to present diagnostic challenges, highlighting the need for additional case reports.

PBL commonly presents with cutaneous breast manifestations that closely resemble other more common types of breast carcinomas or benign lesions, including fibroadenomas, primary breast carcinoma, and ductal carcinoma in situ. In addition to mixed clinical features, radiographic findings may overlap, which can result in delayed diagnosis and treatment.^4^ A definitive diagnosis and subtype classification of PBL requires biopsy followed by confirmatory histopathologic evaluation. Accurate differentiation between PBL and other breast malignancies is crucial, as therapeutic management and prognoses differ substantially.^1^^,^^2^^,^^4^

We present a case of CD5^+^, nongerminal center B-cell (non-GCB)–type DLBCL PBL, which initially manifested as a subcutaneous breast nodule that progressed with increased prominence and associated pruritus. To our knowledge, few cases of CD5^+^ non-GCB DLBCL PBL have ever been reported, with this being the first case isolated to the breast. This case highlights the importance of a thorough evaluation of patient concerns and maintaining a broad differential diagnosis when assessing breast nodules. Additionally, this case adds to the growing body of literature recognizing CD5^+^ non-GCB PBL as a separate clinical entity.

## Case report

A 43-year-old woman with a history of 11-year-old bilateral nontextured silicone breast implants presented to the dermatology clinic for evaluation of a firm, painless subcutaneous nodule on the lateral aspect of the right breast. The patient noted that her implant on the right side had ruptured 3 years prior, and this was repaired by her original surgeon with a bilateral revision with new silicone implants 1 year before her presentation to the clinic. The patient stated that ∼2 days after her revision surgery, a small nodule appeared on the lateral aspect of the right breast. The surgeon determined that imaging was not necessary, and the patient did not undergo further revision of the implant. Approximately 10 months after she first noticed the nodule, it became more prominent, and she also endorsed pruritus. She also noted drenching night sweats 3 to 4 times per week for the past year.

Physical examination revealed a firm, nonmobile, well-circumscribed nodule measuring ∼1.7 × 2 cm in size within the lateral aspect of the right breast at the 11- to 12-o’clock position (Fig 1). Excisional biopsy was performed, and histopathologic analysis revealed partial distortion of the nodal architecture by an atypical lymphoid infiltrate composed of large, atypical cells with oval nuclei, vesicular chromatin, and multiple nucleoli (Fig 2). Immunohistochemical analysis indicated that the atypical cells were positive for CD20, PAX5, CD79a, CD5 (weak), BCL6, MUM1, and BCL2 but negative for CD10, CD30, CD43, ALK1, cyclin D1, and EBER ISH. C-myc was partially expressed (<40%). These findings confirmed a diagnosis of DLBCL, CD5^+^, non-GCB subtype (Fig 3). The patient was referred to oncology for further management and underwent 4 cycles of rituximab, cyclophosphamide, doxorubicin, vincristine, and prednisone therapy. Her most recent positron emission tomography scan reported no evidence of disease.

**Fig 1:**
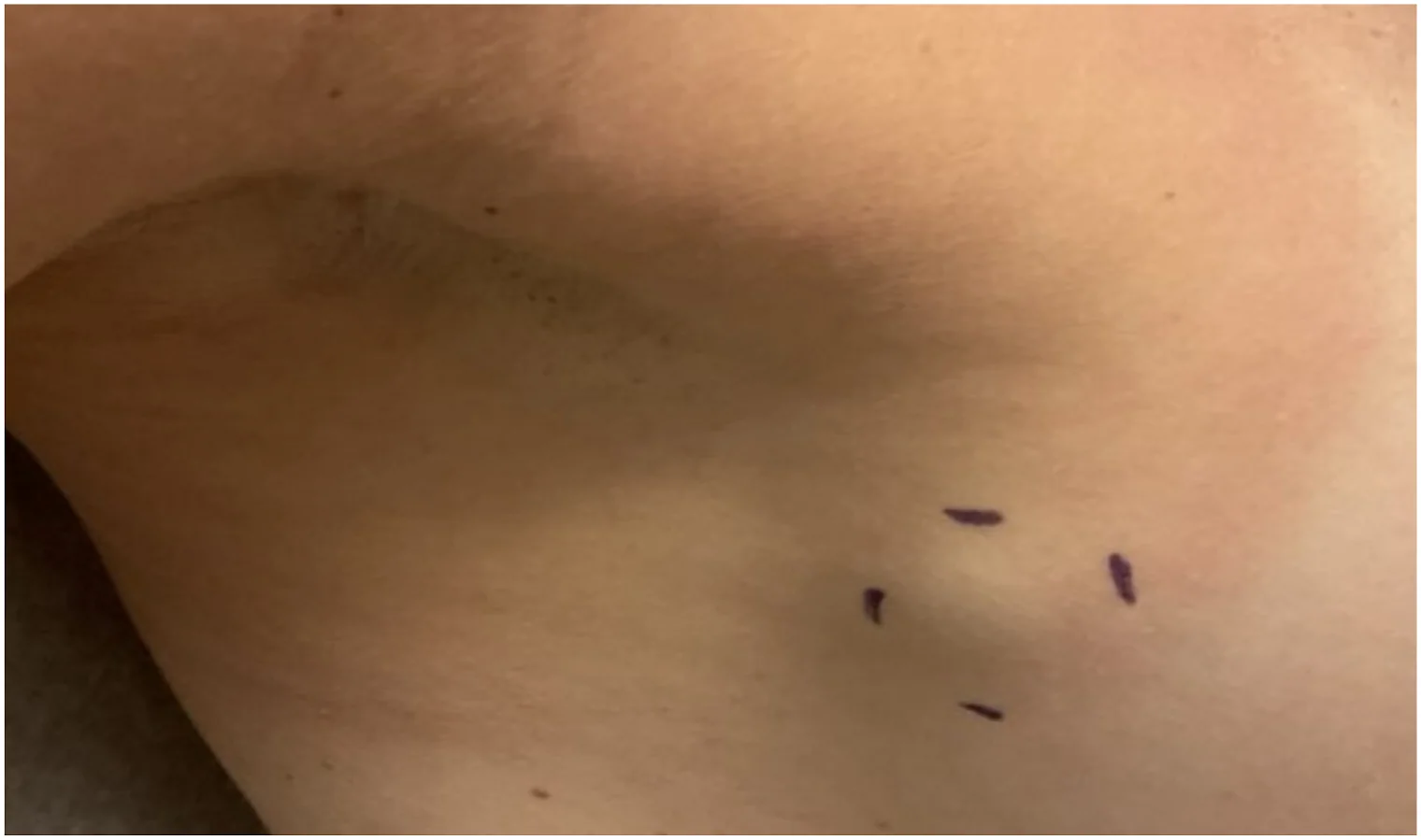
A 1.7 × 2 cm firm, round, subcutaneous nodule within the lateral aspect of the right breast.

**Fig 2:**
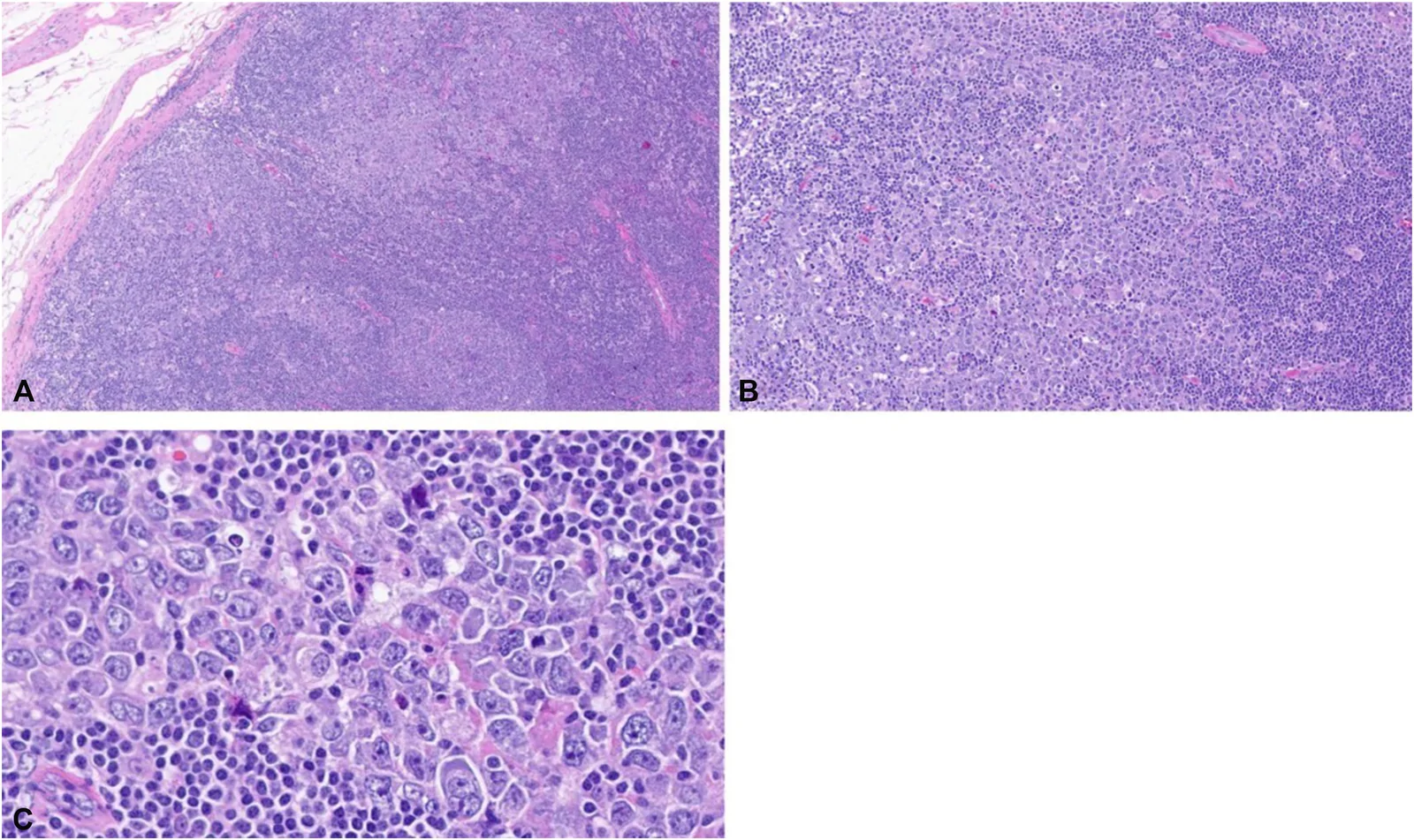
Histopathologic findings of the excisional biopsy of the right breast. **A** and **B**, Nodal architecture is partially distorted by the presence of an atypical lymphoid infiltrate (**A** and **B**, hematoxylin-eosin stain; original magnifications: **A,** ×2; **B,** ×10, respectively). **C**, Atypical lymphoid cells exhibit large, oval nuclei with vesicular chromatin and multiple nucleoli (hematoxylin-eosin stain; original magnification: ×40).

**Fig 3:**
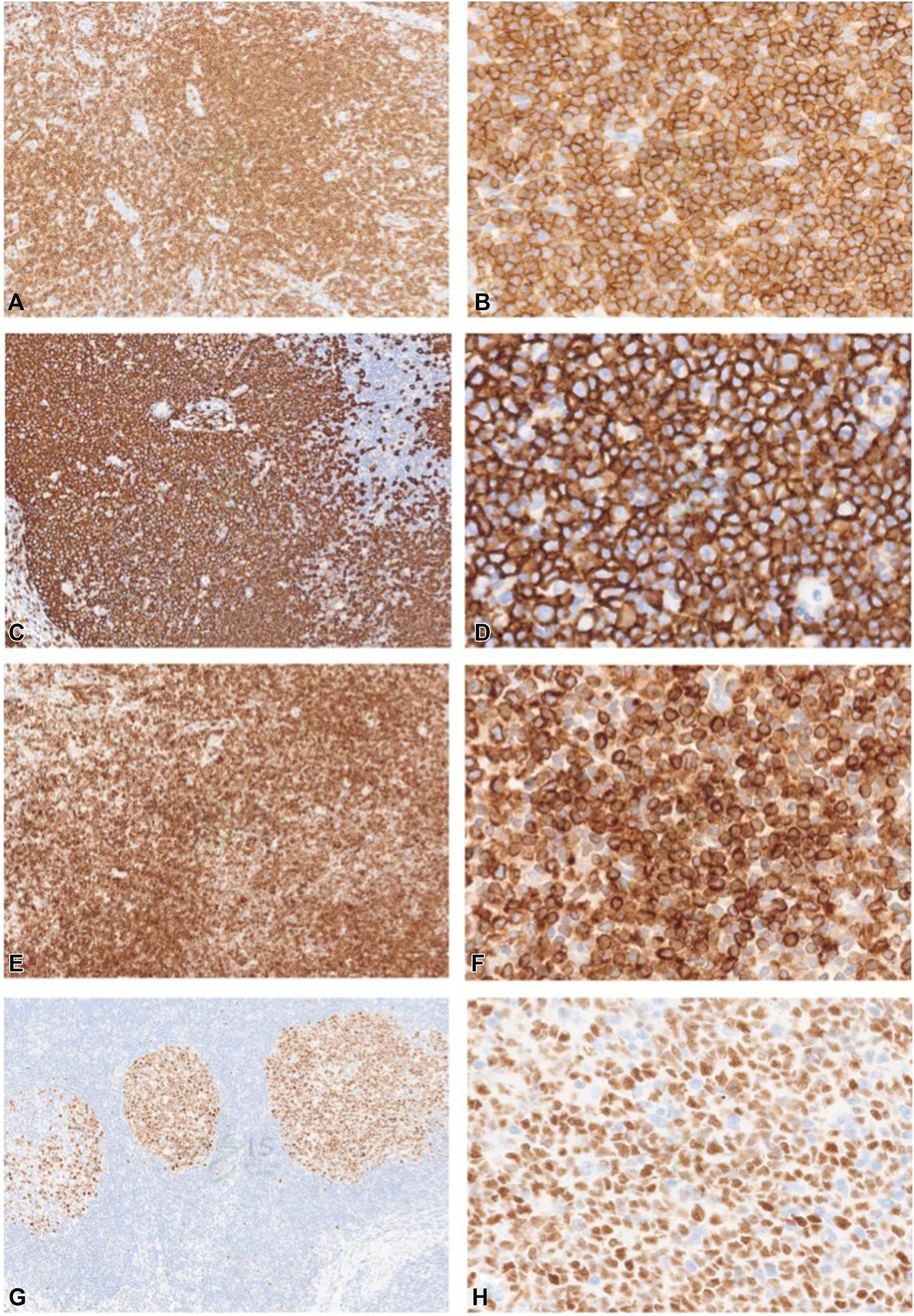
Immunohistochemical staining of the excisional biopsy of the right breast for CD5, CD20, BCL-2, and BCL-6. **A** and **B**, Atypical cells weakly exhibit diffuse membranous CD5 positivity (original magnifications: **A**, ×10; **B**, ×40). **C** and **D**, Immunohistochemistry staining for CD20 is also positive, confirming B-cell lineage (original magnifications: **C**, ×10; **D**, ×40). **E** and **F**, BCL-2 immunohistochemical staining reveals atypical cells with positive cytoplasmic reactivity (original magnifications: **E**, ×10; **F**, ×40). **G** and **H**, BCL-6 staining demonstrates a distinct nuclear staining pattern of the atypical cells (original magnifications: **G**, ×10; **H**, ×40).

## Discussion

PBL is a rare extranodal malignancy, comprising ∼0.04% to 1% of all malignant breast neoplasms. PBL poses significant diagnostic challenges due to its low prevalence and mixed clinical and radiologic features with more common breast pathologies.^1^, ^2^, ^3^, ^4^ DLBCL is the most common subtype of PBL and is characterized by a more aggressive clinical course.^1^, ^2^, ^3^ Approximately 5% to 10% of DLBCLs express CD5, which is a molecule that promotes B-cell survival and provides negative feedback on B-cell receptor signaling, resulting in cell death, increased disease aggressiveness, and a poorer prognosis, as demonstrated in previous studies.^5^, ^6^, ^7^ Few reports have described cases of PBL of the CD5^+^ non-GCB type isolated to the breast, as presented in this case. This case is consistent with existing literature on the rarity and diagnostic complexity of PBL, and it contributes to the growing body of work recognizing CD5^+^ non-GCB PBL as a distinct diagnosis.

Distinguishing PBL from primary breast carcinoma and other breast malignancies is key because treatment approaches differ substantially. Unlike primary breast carcinoma and ductal carcinoma in situ, breast-conserving surgery or radical mastectomy is no longer the preferred treatment for PBL.^2^^,^^8^^,^^9^ In fact, radical mastectomy for PBL has been reported to be associated with higher mortality and poorer clinical outcomes.^2^ Rather, the current standard therapy for PBL, particularly the DLBCL subtype, involves a minimum of 3 cycles, but most commonly 6 cycles of rituximab, cyclophosphamide, doxorubicin, vincristine, and prednisone or cyclophosphamide, doxorubicin, vincristine, and prednisone-like anthracycline-based chemotherapy with rituximab.^1^^,^^2^^,^^4^^,^^8^^,^^10^ Radiation therapy to the ipsilateral breast and lymph nodes after chemotherapy remains controversial, although some studies have reported improved overall survival.^9^ Central nervous system prophylaxis should also be considered because of higher central nervous system recurrence rates in PBL.^2^^,^^8^ Early diagnosis of PBL allows for prompt treatment and reduces the likelihood of unnecessary surgical interventions.

This case highlights the importance of acknowledging and investigating patient concerns with an appropriate evaluation and maintaining a broad differential diagnosis when evaluating cutaneous breast manifestations, particularly in patients presenting with a palpable breast mass and no prior history of lymphoma. PBL should be considered in these patients even with no previous history of lymphoma. As part of the assessment, obtaining a biopsy promptly for histopathologic evaluation with immunochemistry is essential to establish the diagnosis, guide appropriate management, and minimize diagnostic delays or adverse clinical outcomes.

## Conflicts of interest

None disclosed.
